# Soluble fibres modulate dough rheology and gluten structure via hydrogen bond density and Flory-Huggins water interaction parameter

**DOI:** 10.1016/j.crfs.2025.100991

**Published:** 2025-01-30

**Authors:** Stefano Renzetti, Lisa Lambertini, Helene C.M. Mocking-Bode, Ruud G.M. van der Sman

**Affiliations:** a Wageningen Food and Biobased Research, Wageningen University & Research, Bornse Weilanden 9, 6700 AA Wageningen, the Netherlands; b Department of Food, Environmental and Nutritional Sciences (DeFENS), Università Degli Studi di Milano, Via G. Celoria 2, 20133 Milan, Italy

## Abstract

Soluble fibres are gaining increasing interest for functional food applications like bread, but their interaction with gluten and effects on dough rheology are not fully elucidated. This study hypothesized that soluble fibres influence gluten structure and dough rheology by acting as plasticizers and humectants. Plasticizing properties depend on the effective number of hydrogen bonding sites available in the fibre molecule (*N*_*OH,s*_). Humectant properties are related to the water interaction parameter derived from analysis of the sorption behaviour. Oligo-fructoses, inulins, polydextrose and a glucose syrup were added individually and in mixtures to wheat dough to test the hypothesis. PCA and multi-linear regressions showed that the G′ from temperature sweeps increased with an increase in the effective volume fraction of hydrogen bonding sites ($\Phi_{w,eff}$) in the solvent and in the water interaction parameter (*χ*_*eff*_). The enhanced G′ corresponded to a reduction in tan(δ), indicating an increased elastic behaviour. The parameters $\Phi_{w,eff}$ and *χ*_*eff*_ also explained the changes in phase transitions during heating, i.e. T_onset_ and T_peak_ of starch gelatinization (R^2^ > 0.9). Image analysis of the gluten network revealed that fibre structure and physico-chemical properties influenced the gluten network by altering branching rate, lacunarity, and protein strand width. Comparing inulins and polydextrose of similar molecular weights (M_w_) indicated that interactions with gluten were influenced more by *N*_*OH,s*_ than M_w_. High M_w_ inulins, with a linear structure, promoted junctions in the gluten network through hydrogen bonds, and possibly phase separation in gluten-rich and inulin-rich phases. In contrast, the more hydrophilic, branched polydextrose reduced junction formation in the gluten network due to fewer *N*_*OH,s*_. This study provides new insights into the physico-chemical properties of soluble fibres and their role in wheat dough functionality.

## Highlights

• Hydroxyl group density $\Phi_{w,eff}$ modulate phase transitions of fibre-enriched doughs.
• The fibre-water interaction parameter *χ*_*eff*_ and $\Phi_{w,eff}$ modulate dough rheology.
• Fibre structure influences interactions with gluten via hydrogen bonding.
• Large fibres with high number of H-bonding sites increase branching rate of the gluten network.
• Highly branched fibres with low number of H-bonding sites enhance lacunarity of gluten network.

## Graphical abstract

**Figure:**
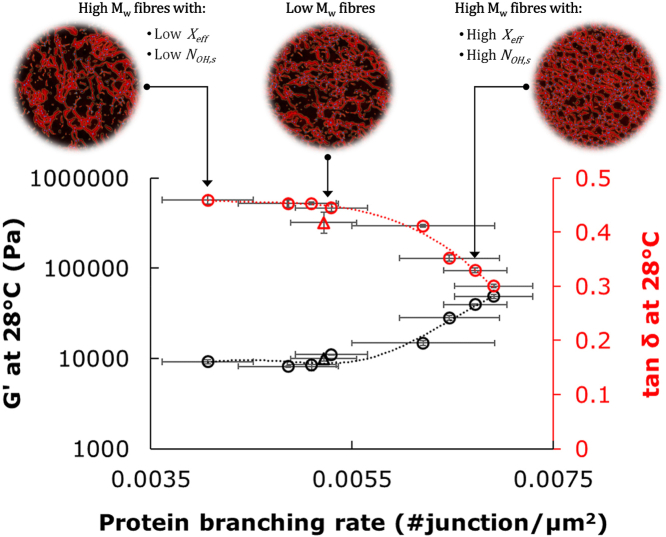

## 1 Introduction

Soluble fibres, such as pectin, arabinoxylan, β-glucans, fructo-oligosaccharides, galacto-oligosaccharides, inulin, and xyloglucans, continue to gain attention due to their health benefits, particularly as fermentable fibres that support gut health (Khorasaniha et al., 2023). When fermented by the gut microbiota, these fibres produce beneficial by-products like short-chain fatty acids, which act as signaling molecules and energy substrates for the host (Abdul Rahim et al., 2019). Specific fibres such as fructo-oligosaccharides, inulin, β-glucans, and arabinoxylan have demonstrated health benefits, reducing risks associated with coronary heart disease, diabetes, and obesity (Guan et al., 2021; Nirmala Prasadi and Joye, 2020). Despite these benefits, dietary fibre consumption in the Western diet remains below the recommended levels of 19–38 g/day based on age and sex (Khorasaniha et al., 2023; Quagliani and Felt-Gunderson, 2017). Recent research emphasizes that oligo- and polysaccharide structures can selectively nourish specific bacteria in the gut microbiome (Rastall et al., 2022), creating opportunities to improve health through targeted functional foods, such as fibre-rich bread products designed for disease prevention. However, incorporating soluble fibres into bread products poses technological challenges due to their interactions with gluten, which can alter gluten aggregation and biopolymer phase transitions, ultimately affecting dough rheology, baking performance, and bread quality (Arufe et al., 2017; Bonnand-Ducasse et al., 2010; Frederix et al., 2004; Wang et al., 2002).

Current research suggests that the effects of soluble fibres on gluten arise through several mechanisms, including water redistribution and partial dehydration of gluten, which can induce changes in gluten's secondary structure (Li et al., 2012; Nawrocka et al., 2017; Zhao et al., 2020; Zhu et al., 2017; Nirmala Prasadi and Joye, 2023). This partial dehydration is often accompanied by a reduction in disulfide bonds within gluten proteins in fibre-enriched dough (Liu et al., 2016; Wang et al., 2017a; Zhou et al., 2014). Additionally, the hydroxyl groups in soluble fibres are believed to facilitate hydrogen bonding with gluten proteins, which increases the proportion of soluble proteins (Nawrocka et al., 2015; Nawrocka et al., 2017a; Nawrocka et al., 2016a; Ribotta et al., 2005; Wang et al., 2020).

The molecular weight (M_w_) of soluble fibres plays a crucial role in these interactions. Low M_w_ fibres exert minimal impact on gluten structure, whereas high M_w_ fibres detrimentally impact gluten aggregation due to their tendency to self-aggregate. This aggregation reduces water availability for gluten and inhibits gluten protein interactions, which can compromise dough properties (Frederix et al., 2004; Huang et al., 2017; Li et al., 2016; Nawrocka et al., 2017a; Wang et al., 2017b). While M_w_ is a known factor influencing fibre-gluten interactions (Frederix et al., 2004; Li et al., 2017; Zhou et al., 2014), a comprehensive understanding of how molecular properties, such as hydrogen-bonding capacity and hygroscopic behaviour, influence these interactions remains incomplete (Zhou et al., 2021).

Among soluble fibres, oligosaccharides (e.g. fructo-oligosaccharides), inulins and polydextrose are particularly relevant in bakery applications for fibre enrichment. Fructo-oligosaccharides and inulins are water-soluble, polydisperse polysaccharides consisting of D-fructose units linked by β-1,2 bonds, with a degree of polymerization (DP) from 2 to 60 (Shoaib et al., 2016). Polydextrose, by contrast, is a synthetic indigestible glucose polymer with randomly linked glucose units and an average DP of 12 (Raninen et al., 2011). Both fibres have been used for fibre enrichment in wheat dough (Ansari et al., 2022; Luo et al., 2017, 2018; Peressini and Sensidoni, 2009), although the physico-chemical mechanisms underlying their effects on dough properties were not fully elucidated and typically attributed to differences in M_w_.

More recently, soluble fibres were studied for their plasticizing and humectant functions in bakery applications (Renzetti and van der Sman, 2024; Renzetti and van der Sman, 2022; van der Sman et al., 2022). The plasticizing role of soluble fibres was described by: i) the volumetric density of hydrogen bonds $\Phi_{w,eff}$ in water solutions which controlled the denaturation and gelatinization temperature of proteins and starches (van der Sman, 2016; Renzetti et al., 2020, 2021), and the viscosity of the solutions (van der Sman and Mauer, 2019), and by ii) the effective number of hydroxyl groups per molar volume of the soluble fibre, i.e. *N*_*OH,s*_*/v*_*s*_, which controlled the pasting properties of starch and flour (Renzetti et al., 2021b; Renzetti, van der Sman, 2022). The hygroscopic behaviour of soluble fibres has been described by the Flory-Huggins water interaction parameter *χ*_*eff*_ (van der Sman, 2017).

Given the influence of $\Phi_{w,eff}$, *N*_*OH,s*_*/v*_*s*_ and *χ*_*eff*_ on biopolymer hydration, water partitioning and phase transitions, this study hypothesized that these parameters also explain the influence of soluble fibres on water absorption by the flour, gluten structure, dough rheology after mixing and during baking, and phase transitions. To test this, oligosaccharides (i.e. fructo-oligosaccharides), inulins, polydextrose, and also a glucose syrup (i.e. Mylose) with M_w_ values ranging from 500 to 3900 g/mol were added at a constant amount to wheat flour, both individually and in binary combinations. Mylose was included due to differences in molecular structure (i.e. glucose units linked by α-1,4 bonds) compared to oligo-fructoses, despite similar M_w_. Inulins and polydextrose with similar M_w_ but different physico-chemical properties were used to distinguish the influence of molecular features. Dough rheology after mixing and during heating and phase transitions were assessed by extensional rheology, dynamic mechanical thermal analysis, and differential scanning calorimetry. Changes in gluten structure were assessed using confocal laser scanning microscopy with image analysis of the gluten network.

## 2 Theoretical background

Following recent developments (Renzetti and van der Sman, 2024; Renzetti and van der Sman, 2022; van der Sman et al., 2022), the working hypothesis of this study was that the effect of soluble fibres on dough rheology after mixing and during heating and on gluten structure is controlled by specific physico-chemical properties of the soluble fibres and of the solvent phase (i.e. water with dissolved fibres): $\Phi_{w,eff}$, *N*_*OH,s*_*/v*_*s*_, and *χ*_*eff*_. The $\Phi_{w,eff}$ describes the plasticizing properties of the solvent which controls biopolymer melting. The $\Phi_{w,eff}$ is computed according to (van der Sman, 2016):

(1) $$\Phi_{w,eff}=\Phi_{w}+\sum_{i}\Phi_{s,i}\;\frac{\;N_{OH,s}v_{w}}{N_{OH,w}v_{s}\;}$$

where $\Phi_{w}$ is the volume fraction of water, $\Phi_{s,i}$ that of the plasticizer and $v_{w}$ and $v_{s}$ are the molar volume of water and plasticizer (i.e. soluble fibres), respectively, obtained from the ratio of their molar weight over their mass density. The *N*_*OH,s*_ represents the number of H-bonding sites effectively available within the fibres, i.e. plasticizer, for intermolecular interactions. For water holds *N*_*OH,w*_ = 2. *N*_*OH,s*_ differs from the total number of hydroxyl groups in a molecule as it is corrected for intramolecular hydrogen bond interactions due to stereochemistry (Pawlus et al., 2012). For plasticizers such as sugars and sugar oligomers, *N*_*OH,s*_ is inversely proportional to the glass transition temperature of the pure compound (Van Der Sman, 2013b):

(2) $$\frac{1}{2}\frac{T_{g\;}-T_{g,w}}{T_{g}^{\infty }-T_{g,w}}=\left(\frac{1}{2}-\frac{N}{N_{OH,s}}\right)$$

Where $T_{g\;}$ is the glass transition temperature of the pure compound, $T_{g,w}$ is the glass transition temperature of pure water (equal to 139 K), $T_{g}^{\infty }$ is the glass transition temperature of a large maltopolymer or starch and equal to 475 K (van der Sman and Mauer, 2019), and $\frac{N}{N_{OH,s}}$ is the inverse of the number of hydroxyl groups per molecule.

From the *N*_*OH,s*_ of sugars and soluble fibres, *N*_*OH,s*_*/v*_*s*_ is obtained where $v_{s}$ is the molar volume of the plasticizer. The *N*_*OH,s*_*/v*_*s*_ parameter is an intrinsic property of the plasticizer, which controls the pasting behaviour of starch and wheat flour as measured by the Rapid Visco Analyzer (Renzetti, van den Hoek et al., 2021; Renzetti, van der Sman, 2022). In the presence of mixtures of plasticizers, *N*_*OH,s*_*/v*_*s*_ is computed from (Renzetti, van den Hoek et al., 2021):

(3) $${\left(\frac{N_{OH,s}}{v_{s}}\right)}_{eff}=\frac{\sum_{i}\Phi_{s}\;\frac{\;N_{OH,s}}{v_{s}\;}}{\sum_{i}\Phi_{s}\;}$$

Where $\Phi_{s}$ is the volume fraction of each plasticizer in the mixture.

The Flory-Huggins water interaction parameter *χ*_*s*_ has been described based on the Flory-Huggins Free Volume theory (FHFV) (Van der Sman, 2013a), including sugars, glucose homopolymers and soluble fibres (van der Sman, 2017; Van Der Sman and Meinders, 2011). For mixtures of sugars and sugar oligomers, it has been shown that the interactions among solutes are about zero (Diarce et al., 2015; Palomo Del Barrio et al., 2016; Van der Sman, 2013a, van der Sman, 2023). Therefore, the water activity (a_w_) of solutions of sugars and sugar oligomer mixtures is controlled by the volume-averaged interaction parameter:

(4) $$\chi_{eff}=\frac{\sum_{i}\Phi_{s,i}∙\chi_{s,i}}{\sum_{i}\Phi_{s,i}}$$

Where *χ*_*s,i*_ is the Flory-Huggins interaction parameter of the solute with water and *Ф*_*s,i*_ is the volume fraction of the solute.

Aside from the properties of the soluble fibres and of the water-fibre mixtures, also variations in the volume fraction of the polymeric components (i.e. gluten and starch) can affect the rheological properties of the dough (Renzetti and van der Sman, 2024). Additionally, the flour amount also affects water distribution. Specifically, it was assumed that the native starch acts as a filler and absorbs only a small amount of water compared to gluten. The volume fraction occupied by the flour, i.e. gluten and starch, was computed from the mass fraction using the mass density *ρ*_*i*_ of water, solutes (sugars and fibres) and protein and starch, as previously described (Renzetti and van der Sman, 2022; Zanoletti et al., 2017).

## 3 Materials and methods

### 3.1 Materials

Wheat flour Edelweiss (15.5% moisture, 10.6% protein, and 71.2% carbohydrates (of which 1.8% sugars, 2.8% dietary fibres and 95.4% starch)) was from Meneba (Rotterdam, the Netherlands), the bakery fat trio puur zacht was from CSM Benelux BV (Goes, the Netherlands). The following glucose syrup and soluble fibres were used in the study: Mylose (Beghin Meiji-Syral, Marckolsheim, France), FOS Actilight 950 P (Beghin Meiji-Syral, Marckolsheim, France), Frutalose OFP (Sensus, Roosendaal, the Netherlands), Frutafit CLR (Sensus, Roosendaal, the Netherlands), Litesse Ultra (Danisco, Redhill, UK), Frutafit IQ (Sensus, Roosendaal, the Netherlands), Frutafit TEX (Sensus, Roosendaal, the Netherlands). To vary the physico-chemical properties of the plasticizers, the fibres were used in single and mixtures. The coding for each fibre is provided in Table 1 together with their physico-chemical properties. Most of the parameters were measured and used in previous studies (van der Sman et al., 2022; Renzetti and van der Sman, 2022; Renzetti, van den Hoek et al., 2021). Only for Frutafit CLR and IQ sorption measurements were required to determine the values of *Χ*_*s*_.

**Table 1:** Structure and physico-chemical properties of the investigated compounds.

Compound	Structure	M_w_ (g/mol)	Density (kg/m^3^)	*T*_*g*_ (K)	*N*_*OH,s*_	*N*_*OH,s/*_*v*_*s*_ (1000 mol/cm^3^)	*Χ*_*s*_
Water	NA	18	1000	139^a^	2	111.1	
Dextrose	NA	180	1540	306^a^	3.98^a^	34.2	0.35^a^
Mylose (Myl)	[αGlcp (1 → 4)]_n_αGlcp (1 → 4)Glc	504	1550	389^a^	7.81^a^	23.2	0.60^a^
FOS Actilight 950 P (FOS)	[βFruf (2 → 1)]_n_βFruf (2 ↔ 1)αGlcp	605	1550	315^b^	4.66^b^	11.9	0.62^b^
Frutalose OFP (OFP)	[βFruf (2 → 1)]_n_βFruf (2 ↔ 1)αGlcp	725	1550	328^a^	5.16^a^	11.0	0.65^a^
Frutafit CLR (CLR)	[βFruf (2 → 1)]_n_βFruf (2 ↔ 1)αGlcp	1769	1550	362^c^	7.24^c^	6.3	0.84^d^
Polydextrose Litesse Ultra (PDX)	α- and β-Glcp (1 → 2), (1 → 3), (1 → 4) and (1 → 6)	2160	1550	353^a^	5.51^a^	4.4	0.80^a^
Frutafit IQ (IQ)	[βFruf (2 → 1)]_n_βFruf (2 ↔ 1)αGlcp	2184	1550	372^c^	8.09^c^	5.7	0.89^d^
Frutafit TEX (TEX)	[βFruf (2 → 1)]_n_βFruf (2 ↔ 1)αGlcp	3877	1550	421^b^	23.18^b^	9.3	1.0^b^

Abbreviations: Fru = fructose; Glc = glucose; p = pyranose ring form; f = furanose ring form; β = beta glycosidic linkage; α = alpha glycosidic linkage.

a From (van der Sman et al., 2022).

b From (Renzetti and van der Sman, 2022).

c From (Renzetti, van den Hoek et al., 2021).

d Determined in this study.

### 3.2 Methods

#### 3.2.1 Moisture sorption behaviour of soluble fibres

The moisture sorption behaviour of the soluble fibres was determined following the method described by (Renzetti et al., 2012). The sorption data were modeled using the FHFV theory described by (Van der Sman, 2013a; van Der Sman and Meinders, 2011). The model requires the dry $T_{g\;}$ and the water interaction parameter *Χ*_*s*_ which were previously reported by (van der Sman et al., 2022) for Mylose, Frutafit OFP and polydextrose Litesse and by (Renzetti and van der Sman, 2022) for FOS Actilight and Frutafit TEX. The dry $T_{g\;}$ for Frutafit CLR and IQ was reported by (Renzetti, van den Hoek et al., 2021). The *Χ*_*s*_ for Frutafit CLR and IQ were obtained in this study by applying the FHFV theory.

#### 3.2.2 Studied dough formulations

Overall, twelve different doughs were prepared, a control dough with wheat flour and eleven doughs with added soluble fibres in single and in mixtures. The formulations and coding for all the doughs are reported in Table 2. The soluble fibres were added in replacement of wheat flour at a fixed amount (i.e. 9%). For each dough formulation, the required water amount to reach a target dough consistency for bread-baking was determined according to ICC standard method 115/1 ICC-Standards, 2006) with few modifications. Briefly, 0.86 g of sodium chloride (Merck, The Netherlands) was added to 50 g of wheat flour or flour–fibre mixtures and pre-mixed for 2 min in a Farinograph-E (Brabender, Duisburg, Germany) equipped with a 50 g mixing bowl. Water addition levels were defined by running an appropriate number of replicates until the maximum dough development was centred on the 420 FU (farinograph units), according to a previously established method (Zanoletti et al., 2017). The optimum water level for each formulation is reported in Table 2. Based on preliminary baking trials, mixing time was kept constant at 9 min for all variations. All dough variations were prepared in triplicates.

**Table 2:** Composition of the studied dough formulations and obtained physico-chemical parameters.

Ingredients (g)	D_Ref	D_FOS	D_Myl	D_OFP	D_Myl_PDX	D_CLR	D_PDX	D_IQ	D_OFP_CLR	D_OFP_TEX	D_CLR_TEX	D_TEX
Wheat flour	86.6	78.8	78.8	78.8	78.8	78.8	78.8	78.8	78.8	78.8	78.8	78.8
Gluten	2.9	2.9	2.9	2.9	2.9	2.9	2.9	2.9	2.9	2.9	2.9	2.9
Salt	1.5	1.5	1.5	1.5	1.5	1.5	1.5	1.5	1.5	1.5	1.5	1.5
Dry yeast	1.0	1.0	1.0	1.0	1.0	1.0	1.0	1.0	1.0	1.0	1.0	1.0
Dextrose	2.0	2.0	2.0	2.0	2.0	2.0	2.0	2.0	2.0	2.0	2.0	2.0
Oil	5.9	5.9	5.9	5.9	5.9	5.9	5.9	5.9	5.9	5.9	5.9	5.9
FOS Actilight 950 P		7.8										
Mylose			7.8		3.9							
Frutalose OFP				7.8					5.3	2.7		
Frutafit CLR						7.8			2.5		1.9	
Litesse Ultra					3.9		7.8					
Frutafit IQ								7.8				
Frutafit TEX										5.1	5.8	7.8
Total	100.0	100.0	100.0	100.0	100.0	100.0	100.0	100.0	100.0	100.0	100.0	100.0

Added water	43.6	35.1	36.4	35.0	35.0	33.8	33.5	33.8	33.9	39.4	39.6	41.6

**Physico-chemical parameters**												
*Ф*_*w,eff*_	0.526	0.485	0.494	0.481	0.482	0.472	0.470	0.472	0.475	0.503	0.503	0.513
*χ*_*eff*_	0.350	0.564	0.548	0.588	0.627	0.739	0.706	0.774	0.636	0.768	0.833	0.865
*N*_*OH,s*_*/v*_*s*_	34.1	21.6	25.5	15.5	18.0	11.9	10.5	11.5	14.4	14.7	13.6	14.2
*Ф*_*flour*_	0.448	0.444	0.438	0.444	0.444	0.450	0.451	0.450	0.449	0.425	0.425	0.417

#### 3.2.3 Thermal transitions in dough by differential scanning calorimetry

Differential Scanning Calorimetry (DSC) was performed using a TA Instruments Q200 type analyzer (TA Instruments, New Castle, DE, USA). About 10 mg of dough was weighed and placed in a high-pressure stainless steel pan. The pan was placed in the DSC together with an empty reference pan and equilibrated at 2 °C for 5 min. After equilibration, the temperature was increased linearly with a rate of 7.5 °C/min from 2 °C to 160 °C. The onset of melting endotherm (T_onset_) and peak temperature (T_peak_) were determined using the analysis tool available in the Universal Analysis software (TA Instruments, New Castle, DE, USA). Experiments were performed in triplicates.

T_onset_ and T_peak_ from the DSC analysis were plotted in the state diagram for bread dough baking, as recently constructed by applying an adapted Flory–Huggins model (Renzetti et al., 2021a,b; Renzetti and van der Sman, 2022; van der Sman et al., 2022). The volumetric density of hydrogen bonds $\Phi_{w,eff}$ in water solutions in each dough was calculated based on the fibres and water contents (Table 2) and the mass densities, molar volumes and hydrogen bonding sites *N*_*OH,s*_ (Table 1), as previously described (Renzetti and van der Sman, 2024).

#### 3.2.4 Dynamic mechanical thermal analysis (DMTA) of dough

DMTA tests were conducted in duplicate according to (Renzetti et al., 2021a) on freshly prepared doughs without yeast. For this, a DHR-2 Rheometer from TA Instruments (New Castle, DE, USA) was used with Peltier plates of parallel geometry and a diameter of 25 mm. Approximately 1 g of dough was placed between plates (loading gap: 20 mm) and compressed until 1.025 mm. Dough excess was removed, silicon oil was applied to prevent sample drying, and the dough was compressed until 1 mm. Before the measurement, the dough was rested for 5 min at 25 °C. Samples were oscillated at a frequency of 1 Hz and heated from 25 to 95 °C with a ramp of 5 °C/min. Once 95 °C was reached, the temperature was kept for 5 min. Before analysis, an oscillation amplitude test was performed from 10^−4^ to 10 to select the linear viscoelastic range. Thus, the strain amplitude was kept at 0.5·10^−3^ for all samples. Key parameters related to physical transitions in the dough were derived from the analysis of the G′ (Pa) and tan(δ) curves in the DMTA by using the analysis functions in TA Trios v3.3 (TA Instruments, New Castle DE, USA): the G′ and tan(δ) value at 28 °C (coded G′_28C_ and tanδ_28C_); the onset temperature, T_on_DMTA, from the evolution of G′ during heating (calculated as the intersection of the tangents of the baseline before the sudden increase in G′ and the tangent of the steep G′ profile after T_onset_); G′ and tan δ at T_on_DMTA (G′_on_ and tanδ_on_); G′ at maximum during heating to 95 °C (G′_max_), tan δ at G′_max_ (tanδ_Gmax_) and the temperature corresponding to G′_max_, i.e., T_Gmax_; G′ and tan(δ) at the end of the holding time at 95 °C (G′_95C_ and tanδ_95C_). The ratio between G′_max_ and the minimum in G’ (abbreviated as G'_max_/G′_min_) was also calculated for each measurement.

#### 3.2.5 Extensional dough rheology with kieffer test

Dough extensibility was tested with a Texture Analyzer (TA-XTplus Texture Analyzer, Stable Micro Sistem, Surrey, UK) equipped with a Kieffer rig. About 10–15 g of dough was placed in a pre-lubricated lower plate of a Teflon mold and compressed with the lubricated top plate. After closing the mold, paraffin oil was placed on the edges to prevent dehydration of the dough. The doughs were left resting at 25 °C for 40 min. Ten dough strips were obtained of which seven were selected based on similarity in shape to be analyzed. Doughs were prepared in triplicates resulting in a total of 21 measurements. Resistance to extension (measured as the maximum force (N) required to break the dough strip), and dough extensibility (expressed as the displacement (mm) until breakage) were obtained from the measurements.

#### 3.2.6 Confocal laser scanning microscopy of dough

To visualize the gluten network in the dough matrix, confocal laser scanning microscopy (CLSM) was performed based on the method of (Renzetti et al., 2021a), with slight modifications. Doughs without yeast were prepared, in which water was partially substituted with a filtered Rhodamine B solution in demi-water (0.1 g/L) to reach a concentration of 1 mg Rhodamine B per 100 g flour. Per dough type, ten images were taken with an LSM 510-META CLSM from Zeiss (Jena, Germany) using 20 × magnification and a resolution of 1024×1024 pixels. To get quantitative results, the images were analyzed with AngioTool64 version 0.6a from the National Cancer Institute (Mary-land, MD, USA) (low threshold: 15; high threshold: 255; vessel thickness: 6, 7, 8, 9; small particles: 10; fill holes: 0 and scaling factor: 0.000440529). Parameters of interest included total protein area, total number of junctions, average protein length, protein branching rate (total number of junctions/total protein area), and average protein width (protein area/total protein length). Each dough variation was prepared in duplicates.

#### 3.2.7 Statistical analysis

Analysis of variance (ANOVA) with Tukey's-Test as post-hoc test at a significance level of *p* < 0.05 was performed with SPSS (IBM, version 25, Chicago, US) to determine whether dough properties were significantly different among the tested formulations. Principal component analysis (PCA) of dough properties was performed with Rstudio (RStudio version 1.1.463, Inc., Boston, MA, USA) using the PCA function of the FactoMineR package (Husson et al., 2022), together with correlation analysis.

Multiple linear regressions were performed using a multivariate linear model with backward selection as recently described (Renzetti and van der Sman, 2024; van der Sman et al., 2022):

$$y=b_{0}+b_{1}∙\Phi_{w,eff}^{*}+b_{2}∙\chi_{eff}^{*}+b_{3}∙N_{OH}^{*}/v+b_{4}∙\Phi_{flour}^{*}$$

where y is the estimated value of the physical properties of dough. $\Phi_{w,eff}^{*}$, $\chi_{eff}^{*}$, $N_{OH}^{*}/v$ and $\Phi_{flour}^{*}$ are reduced variables defined as:

$$\Phi_{w,eff}^{*}=\frac{\Phi_{w,eff}-\Phi_{w,eff}^{\min}}{\Phi_{w,eff}^{\max}-\Phi_{w,eff}^{\min}}$$

$$\chi_{eff}^{*}=\frac{\chi_{eff}-\chi_{eff}^{\min}}{\chi_{eff}^{\max}-\chi_{eff}^{\min}}$$

$$N_{OH}^{*}/v=\frac{N_{OH}/v-N_{OH}^{\min}/v}{N_{OH}^{\max}/v-N_{OH}^{\min}/v}$$

$$\Phi_{flour}^{*}=\frac{\Phi_{flour}-\Phi_{flour}^{\min}}{\Phi_{flour}^{\max}-\Phi_{flour}^{\min}}$$

The superscripts ^*min*^ and ^*max*^ indicate the minimal and maximal values of the variables in the set of dough reformulations. To distinguish the different contributions of variables, the following coefficients were computed:

$$b_{\Phi }=\frac{b_{1}}{\left\|b_{1}\right\|+\left\|b_{2}\right\|+\left\|b_{3}\right\|+\left\|b_{4}\right\|}$$

$$b_{\chi }=\frac{b_{2}}{\left\|b_{1}\right\|+\left\|b_{2}\right\|+\left\|b_{3}\right\|+\left\|b_{4}\right\|}$$

$$b_{N_{OH}/v}=\frac{b_{3}}{\left\|b_{1}\right\|+\left\|b_{2}\right\|+\left\|b_{3}\right\|+\left\|b_{4}\right\|}$$

$$b_{flour}=\frac{b_{4}}{\left\|b_{1}\right\|+\left\|b_{2}\right\|+\left\|b_{3}\right\|+\left\|b_{4}\right\|}$$

From the regression analysis the regression coefficient R^2^, the *p*-value of the variables and of the regression models were reported.

## 4 Results and discussion

### 4.1 Structure and physico-chemical properties of soluble fibres

This study aimed to provide further understanding of the mechanistic principles underpinning the effect of soluble fibres on wheat dough rheology and gluten structure. Specifically, the working hypothesis focused on the role of the effective number of hydrogen bonding sites per fibre (*N*_*OH,s*_) and per unit volume of the fibre (*N*_*OH,s*_*/v*_*s*_), as well as the hygroscopic behaviour described by the water interaction parameter *χ*_*s*_. The structure and physico-chemical properties of the fibres studied are provided in Table 1. Using the physico-chemical properties of the individual fibres, the volumetric density of effective hydrogen bonding sites in the solvent *Ф*_*w,eff*_ (i.e. the water-fibre mixture), the volume-averaged *N*_*OH,s*_*/v*_*s*_ and *χ*_*eff*_ of the fibre mixtures were computed based on the dough formulation (Table 2).

For the fructo-oligosaccharides, an increase in M_w_ and *T*_*g*_ corresponded to a higher *N*_*OH,s*_. Notably, the glucose syrup Mylose showed a higher *N*_*OH,s*_ than FOS and OFP due to its high *T*_*g*_, despite its lower M_w_ compared to these fructo-oligosaccharides. Polydextrose exhibited a *N*_*OH,s*_ similar to that of FOS, despite having a high M_w_ similar to that of inulin IQ. This relatively low number of effective hydrogen bonding sites in polydextrose was likely due to its branched structure compared to the other fibres in this study.

**Fig. 1:**
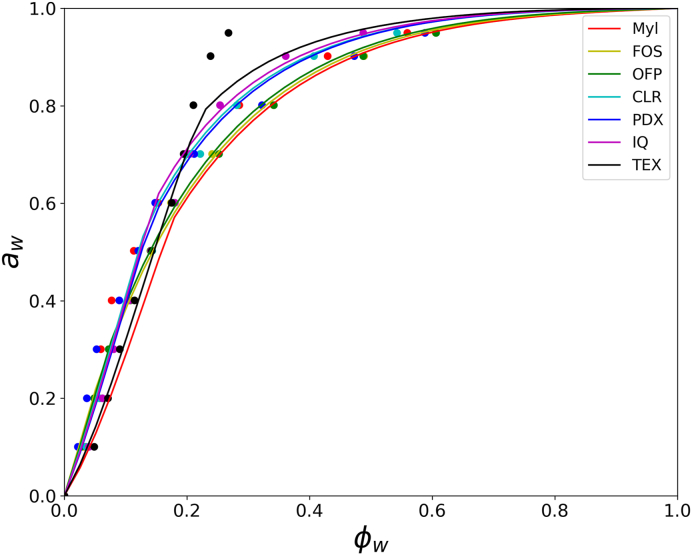
Moisture sorption of solubre fibres in the study as determined at 25 °C. Filled circles represent experimental data. The continuous lines represent the prediction using the FHFV model. The glucose syrup and oligosaccharides Myl, FOS and OFP are the most hygroscopic, with the water interaction parameter *Χ*_*s*_ being 0.60, 0.62 and 0.65, respectively (the lower *Χ*_*s*_ the higher the interaction with water). CLR, PDX, IQ and TEX are less hygroscopic than the oligosaccharides with *Χ*_*s*_ being 0.84, 0.80, 0.89 and 1.0, respectively.

The water interaction parameter *χ*_*s*_ of the fibres was obtained by modeling their sorption isotherm using the FHFV theory. The FHFV equation well described the sorption isotherm data of the soluble fibres, with some deviations observed primarily for TEX (Fig. 1). The deviations for TEX may be related to the formation of crystalline domains, as the fibre is not completely soluble in water. In such cases, the crystalline domains do not absorb water and the effective volume fraction of water $\Phi_{w}$ in the amorphous fraction is higher than what was reported in the experimental data. The samples Myl, FOS and OFP showed better humectant properties than CLR, IQ and PDX, as reflected by their lower χ_s_ (Table 1). This finding was expected since *χ*_*s*_ is mainly a function of M_w_ for sugars and sugar oligomers (van der Sman, 2017). However, the highly branched structure of polydextrose enabled better humectant properties than similarly M_w_ fibres like CLR and IQ. With the highest M_w_ among the fibres studied, TEX was also the least hygroscopic. When the *T*_*g*_ of the fibre-water mixture is lower than the measuring temperature *T*, the sorption behaviour is entirely related to *χ*_*s*_. For all fibres in this study, *T = T*_*g*_ occurred at a maximum volume fraction of water $\Phi_{w}$ of 0.2. Therefore, the humectant properties of the fibres in the studied dough formulations were fully described by *χ*_*s*_.

### 4.2 Effect of soluble fibres on the water absorption of flour

In general, the addition of the soluble fibres reduced the amount of added water required compared to the reference dough, based on farinograph analysis (Table 2), while the a_w_ remained within a limited range (data not shown). Similar reductions in added water with addition of soluble fibres have been previously observed for inulin-enriched dough (Luo et al., 2018; Peressini and Sensidoni, 2009), and were explained by the plasticizing role of low M_w_ components like sugars and oligosaccharides (Rouillé et al., 2005). However, water addition of the doughs in this study could not be explained solely by the M_w_ of fibres. Mylose, FOS and OFP had lower M_w_ and higher plasticizing function than CLR, PDX and IQ, as described by the number of H-bonding sites per molar volume *N*_*OH,s*_*/v*_*s*_ (Table 1), but resulted in slightly higher water addition in the dough (Table 2 and Fig. S1 supplementary). The soluble fibres could exert both plasticizing and humectant functions. The humectant behaviour of the fibre mixture is described by χ_eff_ (or by χ_s_ for individual fibres), where a higher χ_eff_ indicates lower affinity for water (Table 1). High M_w_ fibres are generally less hygroscopic than those with low M_w_. Therefore, for similar water and fibre contents, gluten is less hydrated in the presence of fructo-oligosaccharides of low M_w_ than with inulins. Assuming that, at the concentration used, the soluble fibre do not phase separate, the water addition level determined with the farinograph was most likely the result of the interplay between: i) the plasticizing behaviour of the fibres, which is higher for low M_w_ oligosaccharides as described by *N*_*OH,s*_*/v*_*s*_ (Table 1), and ii) competition for water with gluten, caused by the high water affinity of the low M_w_ fibres (De Gennaro et al., 2000). Additionally, high M_w_ inulins like TEX can also phase separate, resulting in a fibre-rich phase that binds water (Kawai and Hagura, 2012; Renzetti, van den Hoek et al., 2021; Renzetti and van der Sman, 2024). Under these conditions, more water was required in the TEX-enriched dough for gluten hydration compared to the other variations in the study (Table 2).

### 4.3 Effect of soluble fibres on phase transitions in wheat dough

**Fig. 2:**
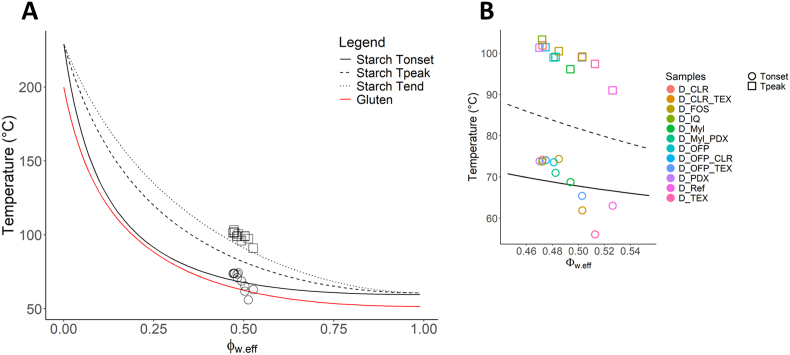
T_onset_ and T_peak_ of starch gelatinization as determined by DSC analysis for the dough samples in Table 2 and plotted in the supplemented state diagram for dough baking with phase transitions for gluten and starch (A). The T_onset_ and T_peak_ obtained for the dough samples are specified with colour codes (B). The state diagram is adapted from (Renzetti et al., 2021a, Renzetti et al., 2021b).

One of the hypotheses of the study was that phase transitions related to starch gelatinization in wheat dough are controlled by the effective volume fraction *Ф*_*w,eff*_ of the water-fibres mixture. The *Ф*_*w,eff*_ is determined by the amount of water added and the contribution of the fibres to the number of hydrogen bonds in the solution. The phase transitions involved in bread baking are presented in the state diagram of Fig. 2, showing the importance of gluten thermosetting and starch gelatinization in forming the solid crumb structure. The phase transitions are based on Flory-Huggins theory for polymer melting as applied to gluten and starch (van der Sman and Renzetti, 2019; Renzetti et al., 2021a,b).

The state diagram is plotted assuming equal partitioning of water between gluten and starch, due to the similar sorption isotherms (van der Sman et al., 2022). The melting transitions obtained from DSC analysis of the doughs were related to starch gelatinization (Supplementary Table S1) and plotted in the state diagram using *Ф*_*w,eff*_. As hypothesized, the T_onset_ and T_peak_ of starch gelatinization were functions of *Ф*_*w,eff*_, consistent with recent studies (Renzetti, van den Hoek et al., 2021; van der Sman and Mauer, 2019). The T_onset_ aligned well with the predictions from the state diagram, while T_peak_ showed higher temperatures than predicted, approaching the end temperature of starch gelatinization.

### 4.4 Effect of soluble fibres on the thermo-mechanical behaviour and extensional rheology of wheat doughs

The working hypothesis of this study was that the physico-chemical parameters *Ф*_*w,eff*_, χ_eff_ and *N*_*OH,s*_*/v*_*s*_ would largely control dough rheology after mixing and during heating. Additionally, the volume fraction of flour ${\;\Phi }_{flour}$ was also considered due to the differences in the farinograph water absorption among the formulations. Significant differences were observed in the DSC analysis, DMTA and Kieffer rig test among the studied variations (Supplementary Tables S1 and S2; Supplementary Figs. S2 and S3).

**Fig. 3:**
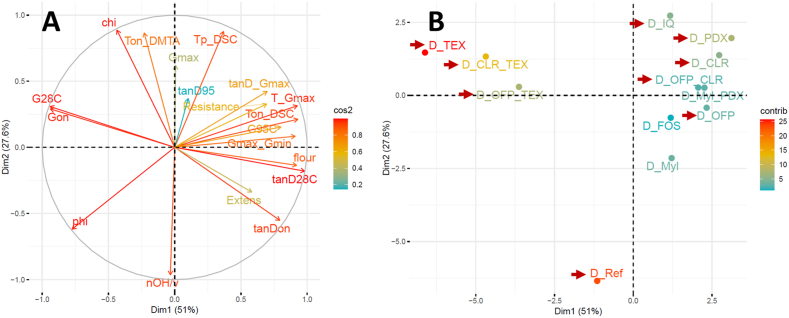
PCA with loading plot of physico-chemical, rheological parameters and phase transitions of dough (A) and sample scores (B). Sample codes are explained in Table 2. In the PCA plots, the physico-chemical parameters are: phi = $\Phi_{w,eff}$, chi = χ_eff_, nOH_v = N_OH,s_/v_s_ and phi_flour = ${\;\Phi }_{flour}$. Measured parameters are: Ton_DSC = onset temperature of starch gelatinization from DSC of dough, Tp_DSC = peak temperature of starch gelatinization from DSC of dough, Ton_DMTA = onset temperature of structure formation in DMTA, T_Gmax = T_peak_ corresponding to max in G′ after T_onset_, G28C = G′ at 28 °C, Gon = G′ at T_onset_ in DMTA, Gmax = max in G′ after T_onset_ in DMTA, G95C = G′ at 95 °C, Gmax_Gmin = Ratio of G'_max_ and G'_min_, tanD28C = tan(δ) at 28 °C, tanDon = tan(δ) at T_onset_ in DMTA, tanD95 = tan (δ) at 95 °C, tanD_Gmax = tan(δ) at G'_max_, Resistance = Resistance to extension, Extens = Maximum extensibility. The red arrows indicate samples selected for CLSM analysis of gluten structure.

**Fig. 4:**
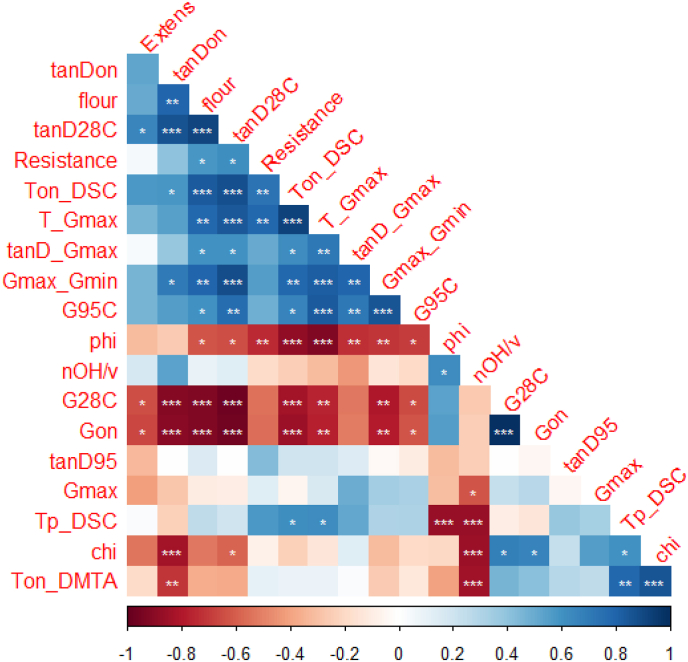
Correlation analysis of physico-chemical parameters, dough rheology and phase transitions. The physico-chemical parameters are: phi = $\Phi_{w,eff}$, chi = χ_eff_, nOH_v = N_OH,s_/v_s_ and phi_flour = ${\;\Phi }_{flour}$. Measured parameters in the dough are: Ton_DSC = onset temperature of starch gelatinization from DSC of dough, Tp_DSC = peak temperature of starch gelatinization from DSC of dough, Ton_DMTA = onset temperature of structure formation in DMTA, T_Gmax = T_peak_ corresponding to the max in G′ after T_onset_, G28C = G′ at 28 °C, Gon = G′ at T_onset_ in DMTA, Gmax = max in G′ after T_onset_ in DMTA, G95C = G′ at 95 °C, Gmax_Gmin = Ratio of G'_max_ and G'_min_, tanD28C = tan(δ) at 28 °C, tanDon = tan(δ) at T_onset_ in DMTA, tanD95 = tan(δ) at 95 °C, tanD_Gmax = tan(δ) at G'_max_, Resistance = Resistance to extension, Extens = Maximum extensibility. Statistical analysis: ∗*p* < 0.05, ∗∗*p* < 0.01, ∗∗∗*p* < 0.001.

PCA analysis was performed to better understand the influence of the physico-chemical parameters on thermal transitions and dough rheology (i.e. thermo-mechanical behaviour and extensional rheology) (Fig. 3). The first two principal components accounted for almost 79% of the total variance, providing a very good representation of the differences among samples. PC_1_ accounted for 51% of the variance, which on the right-hand side was mainly associated to the onset of starch gelatinization (T_on_DSC), the tan(δ) parameters (i.e. tan(δ)_28C_, tan(δ)_on,_ tan(δ)_Gmax_), G'_max_/G'_min_ and the extensional properties (i.e. resistance to extension and extensibility). These properties were significantly correlated positively with ${\;\Phi }_{flour}$ and inversely with *Ф*_*w,eff*_ (*p* < 0.05), as confirmed by correlation analysis (Fig. 4). On the left-hand side of PC_1_, the main parameters were G′_28C_ and G_on_, which were also significantly correlated with ${\;\Phi }_{flour}$ and *Ф*_*w,eff*_. Conversely, PC_2_ was mainly associated with the onset of structure formation in the DMTA (i.e. T_on_DMTA) and the peak temperature of starch gelatinization T_p_DSC. These parameters were positively correlated with *χ*_*eff*_ and inversely with *N*_*OH,s*_*/v*_*s*_ (Fig. 4). In general, the addition of soluble fibres resulted in an increase in T_p_DSC and T_on_DMTA compared to the reference dough (Fig. 3B), with effect size influenced by the hygroscopic behaviour of the fibres (Fig. 4). A clear distinction between fibres was observed with regards to the influence on dough rheology. Formulations containing inulins IQ and TEX (i.e with the largest M_w_), either alone or in combination with another fibre, increased G′_28C_ and G_on_ and concomitantly decreased the corresponding tan(δ) compared to the reference dough (Fig. 3 and Supplementary Table S1). Conversely, all other fibres showed G′ and tan(δ) similar to the reference. Regarding extensional behaviour, all doughs containing TEX did not affect the resistance to extension, unlike other fibres which increased resistance (Fig. 3 and Supplementary Table S2). Dough extensibility was significantly reduced with the addition of IQ and TEX.

The G′ and tan(δ) for cereal-based doughs are strongly influenced by the water-holding capacity of the constituent ingredients, with G′ and tan(δ) inversely related to each other (van der Sman et al., 2024). Except for high M_w_ inulins (e.g. TEX), which can form gels under shear or heating conditions at low concentrations (Kim et al., 2001; Mensink et al., 2015), soluble fibres would not hold water but can modulate the partitioning of the moisture in the dough. The inverse relation between G′ and tan(δ) and their correlation with *Ф*_*w,eff*_ suggest the importance of gluten hydration in the rheological behaviour of the dough at small deformations. The plasticizing ability of the solvent (i.e water with dissolved fibres), as described by *Ф*_*w,eff*_, is important for gluten development during mixing. The increase in G’ (and conversely lower tan(δ)) after mixing with increasing *Ф*_*w,eff*_ suggested enhanced gluten development by promoting cross-linkages in the network. Similarly, the extent of network formation during baking represented by the G'_max_/G'_min_ parameter (Arufe et al., 2017), was inversely related to *Ф*_*w,eff*_. A high G'_max_/G'_min_ resulting from low *Ф*_*w,eff*_ indicated a high sensitivity of the dough to the thermal process due to limited gluten development. Conversely, an increase in the solvent's plasticizing ability, i.e. high *Ф*_*w,eff*_, provided a reduction in G'_max_/G'_min_ due to extensive cross-linking of gluten already after mixing, thus reducing the dough's thermal sensitivity (Arufe et al., 2017).

The different effects observed between the small M_w_ fibres and the formulations containing TEX (and mixtures thereof) and IQ, suggested a specific mechanism related to the physico-chemical properties of the fibres. The increase in G′ and concomitant decrease in tan(δ) observed with the large M_w_ fibres aligned with previous findings from (Peressini and Sensidoni, 2009), attributed to direct gluten-inulin interactions (Wang et al., 2002). The results of this study further suggest that the high effective number of hydrogen bonding sites available with large inulins like IQ and TEX (Table 1) may have favoured stable hydrogen bond interactions with gluten that affected its structure. Additionally, high concentrations (about 18–21% in water in this study) of high M_w_ inulins can induce phase separation into gluten-rich and inulin-rich phases, increasing protein aggregation (Guo et al., 2021). Conversely, PDX with a similar M_w_ to IQ but a lower number of effective hydrogen bonding sites (Table 1) did not stiffen the dough. PDX-enriched dough showed rheological properties similar to the doughs with oligo-fructose and low M_w_ inulins. This result suggests that stable and extensive hydrogen bond interactions between fibres and gluten may be driven more by the effective number of hydrogen bonding sites (*N*_*OH,s*_), which is influenced by the fibre's structure rather than by M_w_.

### 4.5 Quantitative relations between dough rheology and physico-chemical parameters

Multilinear regression analysis was performed to better understand the contribution of the physico-chemical parameters on the thermo-mechanical and extensional properties of the dough (Table 3). In general, phase transitions and dough rheology were largely controlled by the combination of *Ф*_*w,eff*_, *χ*_*eff*_, and *N*_*OH,s*_*/v*_*s*_, and in most cases by *Ф*_*w,eff*_ and *χ*_*eff*_. These results confirmed that the plasticizing properties of the solvent (water with dissolved fibres) and moisture distribution were the main mechanisms controlling dough properties. In agreement with these findings, the partitioning of water among the hydrophilic components of the dough (i.e. starch, gluten, fibers, and sugar) is recognized to be of key importance for dough development and rheology. The effect of flour composition on moisture distribution has been practically assessed using methodologies like the solvent retention behaviour (Kweon et al., 2011). The presence of several hydrophilic components results in competition for water during mixing, with the amount of each individual component affecting moisture partitioning and gluten hydration. As extensively reviewed by (Zhou et al., 2021), the main mechanisms driving the effect of soluble fibres on gluten development is related to the redistribution of water and non-covalent interactions via hydrogen bonds. The parameters *Ф*_*w,eff*_ and *χ*_*eff*_ provide quantitative information on those mechanisms by describing the plasticizing properties of the solvent (i.e. the volumetric density of effective hydrogen bonding sites in the water-fibre mixture) and the hygroscopic properties of the fibres, which in turn control dough rheology and phase transitions during baking (van der Sman et al., 2022).

**Table 3:** Multi-linear regression on DSC and DMTA data from dough analysis as controlled by physico-chemical parameters. Dough properties showing significant variations (*p* < 0.05) are reported.

Symbols	Description	R^2^	*b*_*Φ*_	*b*_*χ*_	*b*_*NOH/v*_	*b*_*lour*_	p_Φ_	p_χ_	p_NOH/v_	p_flour_	p_tot_
DMTA and DSC											
T_on_DSC	T_onset_ starch gelatinization	0.957	−0.66	−0.34			<0.001	<0.001			<0.001
T_p_DSC	T_peak_ starch gelatinization	0.933	−0.60	0.40			<0.001	<0.001			<0.001
T_on_DMTA	T_onset_ structure setting in DMTA	0.805	−0.52			−0.48	<0.001			<0.001	<0.001
T_Gmax_	T_peak_ corresponding to max in G′ after T_onset_	0.958	−0.72		0.28		<0.001		<0.001		<0.001
G’_28C_	G’ at 28 °C	0.946	0.43	0.57			<0.001	<0.001			<0.001
G'_on_	G’ at T_onset_ in DMTA	0.947	0.44	0.56			<0.001	<0.001			<0.001
G'_max_	max in G′ after T_onset_ in DMTA	0.392			−1.00				0.029		0.029
G’_95C_	G’ at 95 °C	0.478	−1.00				0.013				0.013
G'_max_/G'_min_	Ratio of G'_max_ and G'_min_	0.739	−0.60	−0.40			0.010	0.22			0.002
tan(δ)_28C_	tan(δ) at 28 °C	0.969	−0.48	−0.52			<0.001	<0.001			<0.001
tan(δ)_on_	tan(δ) at T_onset_ in DMTA	0.885	−0.30	−0.70			0.004	<0.001			<0.001
tan(δ)_95C_	tan(δ) at 95 °C	0.000									
tan(δ)_Gmax_	tan(δ) at G'_max_	0.647		0.45		0.55		0.025		0.003	0.009
Extensional properties											
Resistance	Resistance to extension	0.817	−0.33	0.26	0.41		<0.001	0.045	0.017		0.003
Extens	Maximum extensibility	0.588		−0.59	−0.41			0.007	0.031		0.018

### 4.6 Effect of soluble fibres on the microstructure of the gluten network

**Fig. 5:**
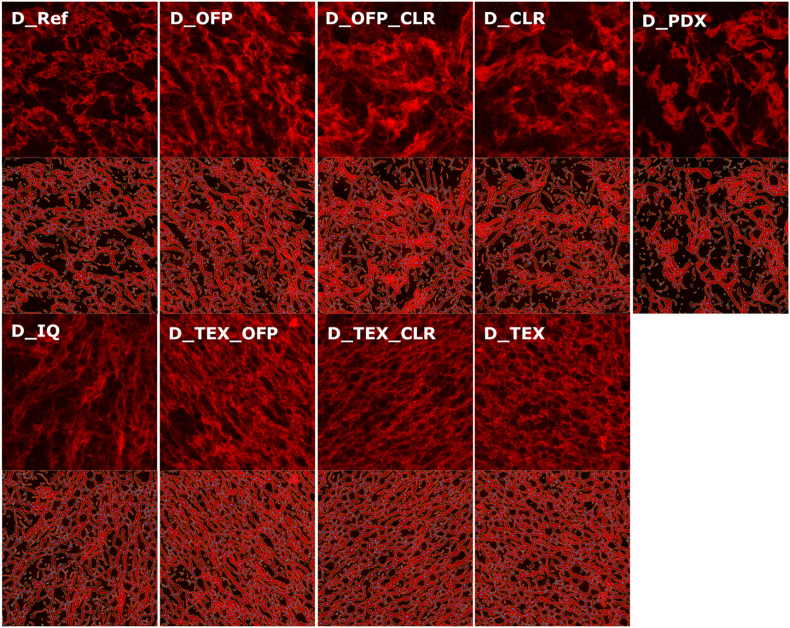
Confocal laser scanning microscopy of gluten network for the different doughs investigated with protein stained in red (images with sample codes). Images were obtained with 20× magnification. For each CLSM image, the result of the protein network analysis with AngioTool is provided below, with junctions shown in blue, protein skeleton shown in red, and protein outline shown in yellow.

The effect of soluble fibres on gluten structure was studied with CLSM on nine samples selected based on PCA analysis of dough rheology (Fig. 3). Image analysis of the gluten network was also performed to quantify structural features. The CLSM images collected for the nine dough formulations are shown in Fig. 5. A homogeneously distributed protein network characterized by gluten strands was observed in the reference dough. Unstained areas appearing in black were likely filled with interspersed starch granules and air bubbles. The addition of small M_w_ fibres like OFP and CLR did not seem to substantially alter the gluten structure, but areas with more protein-rich spots were observed concomitantly with some larger unstained areas. These changes were more evident with the addition of PDX, a high M_w_ and highly branched fibre, as proteins seemed less homogeneously distributed and rather concentrated in certain areas, leaving large spots for starch granules and air bubbles. On the contrary, the addition of high M_w_ fibres (i.e. IQ, TEX and mixtures containing TEX) resulted in a gluten network that seemed even more homogeneously distributed than the reference dough and with an increased amount of interconnections among gluten strands, providing a network with a finer mesh size than the reference. A fine mesh gluten network in the presence of high M_w_ inulin was also reported by (Peressini and Sensidoni, 2009).

The visual observations in this study were well supported by image analysis of the gluten network, providing quantitative information of its structural features (Table 4). The number of junctions in the protein network and the branching rate significantly increased for the doughs containing IQ, TEX and mixtures including TEX compared to the reference. In contrast, a significant decrease in the number of junctions and branching rate was observed for PDX-enriched dough.

**Table 4:** Gluten microstructural parameters as derived from analysis of CLSM images.

Samples	Protein area (μm^2^)	Number of Junctions	Number of End Points	Total Protein Length (μm)	Average Protein Length (μm)	Lacunarity	Branching rate	Endpoint rate	Protein width (μm)
D_Ref	91909^cd^	480^c^	418^cd^	14200^b^	189^cd^	0.087^bc^	0.0052^b^	0.0046^cde^	6.47^bc^
D_OFP	92412^cd^	470^c^	510^a^	13686^b^	134^d^	0.088^bc^	0.0051^b^	0.0056^bc^	6.75^ab^
D_CLR	87774^d^	429^c^	500^ab^	13182^b^	120^cd^	0.102^b^	0.0049^bc^	0.0057^b^	6.68^ab^
D_OFP_CLR	94785^bcd^	502^c^	517^a^	14160^b^	138^cd^	0.083^bcd^	0.0053^b^	0.0055^bc^	6.70^ab^
D_PDX	73117^e^	299^d^	541^a^	10386^c^	60^d^	0.154^a^	0.0041^c^	0.0074^a^	7.05^a^
D_IQ	97692^abc^	609^b^	465^abc^	15609^a^	212^bc^	0.074^cd^	0.0062^a^	0.0048^bcd^	6.26^c^
D_OFP_TEX	104725^a^	678^ab^	429^bcd^	16632^a^	313^ab^	0.062^d^	0.0065^a^	0.0041^de^	6.30^c^
D_CLR_TEX	102554^ab^	690^ab^	397^cd^	16711^a^	357^a^	0.066^cd^	0.0067^a^	0.0039^de^	6.14^c^
D_TEX	105060^a^	726^a^	381^d^	17024^a^	396^a^	0.061^d^	0.0069^a^	0.0036^e^	6.17^c^
*p*	<0.0001	<0.0001	<0.0001	<0.0001	<0.0001	<0.0001	<0.0001	<0.0001	<0.0001

The branching rate describes the number of junctions for the determined protein area, thus providing a structural feature that is independent of the specific image section used (Bernklau et al., 2016). The rate of open-ended protein threads (i.e. end-point rate), which can be used as an interpretation of network cohesion (i.e. high rate indicating low cohesion) (Bernklau et al., 2016), was also significantly increased with the addition of PDX compared to the reference dough. Protein width was the highest for the dough with PDX, thus confirming the visual observation of protein-rich areas with fewer gluten strands. Lacunarity provides quantitative information on irregularities in the protein network and the presence of gaps. The higher the values for lacunarity, the higher the irregularities in the network structure. Doughs containing IQ, TEX and mixtures with TEX showed lower lacunarity than the reference dough, thus confirming the visual observation of a more homogeneous gluten structure. On the contrary, dough with PDX showed a significantly higher lacunarity than the reference dough (Table 4).

### 4.7 Correlation between dough rheology and gluten microstructure

**Fig. 6:**
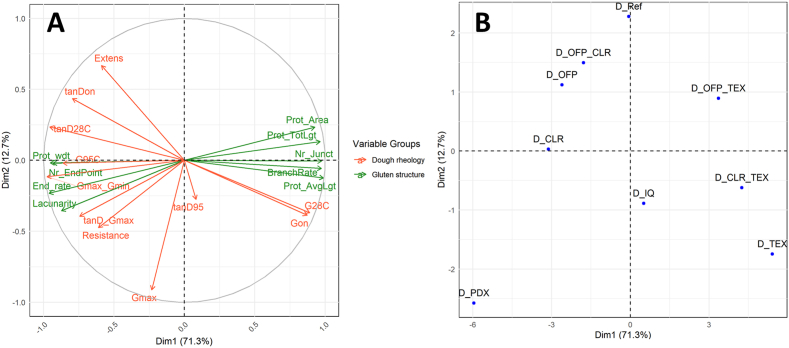
PCA with loading plot of rheological properties and gluten network parameters from Angiotool software (A) and sample scores (B). Sample codes are explained in Table 2. In the PCA plots, the rheological and gluten network parameters are: G28C = G′ at 28 °C, Gon = G′ at T_onset_ in DMTA, Gmax = max in G′ after T_onset_ in DMTA, Gmax_Gmin = Ratio of G'_max_ and G'_min_, tanD28C = tan(δ) at 28 °C, tanDon = tan(δ) at T_onset_ in DMTA, tanD_Gmax = tan(δ) at G'_max_, Resistance = Resistance to extension, Extens = Maximum extensibility, Prot_Area = Protein area in μm^2^, Nr_Junct = Number of Junctions, Prot_TotLgt = Total Protein Length, Prot_AvgLgt = Average Protein Length, Nr_EndPoint = Number of End Points, Lacunarity = Lacunarity, BranchRate = Branching rate, EndRate = End point rate, Prot_wdt = Protein width.

**Fig. 7:**
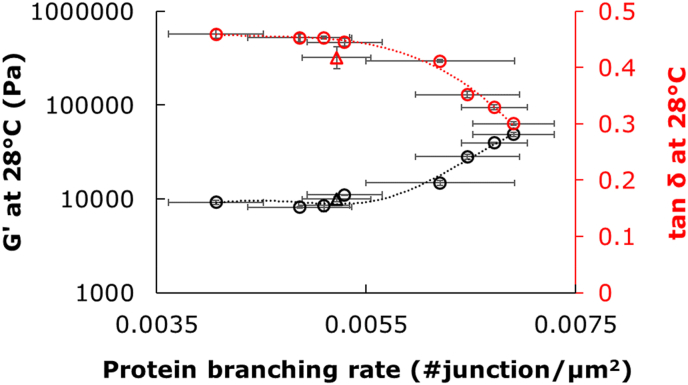
G′ and tan(δ) as obtained at 28 °C during DMTA measurement of the different dough samples plotted against the protein branching rate as determined by image analysis of the protein network. Open triangles represent the reference dough without fibres; open circles represent the fibre-enriched dough samples.

PCA and correlation analysis were performed to relate the microstructural features of gluten to the rheological properties of the doughs. The PCA explained 84% of the total variance, with PC_1_ contributing to almost 72% (Fig. 6). The G′ and tan(δ) parameters extracted from DMTA analysis were largely associated with PC_1_ together with the microstructural features. All DMTA parameters, except for G_max_ and tan(δ)_95C_, were highly correlated with several of the microstructural features (Table 5). Resistance to extension correlated with the number of endpoints in the gluten threads and with the endpoint rate, while no correlations were found for extensibility. The addition of high M_w_ fibres like TEX and IQ enhanced G′ and lowered tan(δ) compared to the reference dough, which was associated with an increase in structural features like the number of junctions, branching rate, average protein length and a reduction in lacunarity (Fig. 6 and Table 5). As shown in Fig. 7, increasing branching rate (i.e. above 0.055 junctions per μm^2^) resulted in substantial enhancement of G’ with a concomitant reduction in tan(δ). Conversely, PDX showed opposite effects on the number of junctions, branching rate, average protein width and lacunarity compared to IQ and TEX, despite a high M_w_ (Fig. 6). These results confirmed that the specific physico-chemical properties of the fibres, i.e. the effective number of hydrogen bonding sites *N*_*OH,s*_ and the water interaction parameter *χ*_*s*_, modulate gluten structure rather than M_w_.

**Table 5:** Pearson's correlation between structural parameters of gluten network and rheological properties.

Symbols	G’_28C_	G_on_	G_max_	G’_95C_	G'_max_/G'_min_	tan(δ)_28C_	tan(δ)_on_	tan(δ)_95C_	tan(δ)_Gmax_	Resistance	Extension
Prot_Area	0.72∗	0.69∗		−0.71∗	−0.91∗∗∗	−0.80∗∗	−0.69∗		−0.72∗		
Nr_Junct	0.85∗∗	0.85∗∗		−0.78∗	−0.94∗∗∗	−0.91∗∗∗	−0.81∗∗		−0.68∗		
Nr_EndPoint	−0.82∗∗	−0.82∗∗		0.91∗∗∗	0.93∗∗∗	0.91∗∗∗			0.71∗	0.7∗	
Prot_TotLgt	0.78∗	0.75∗		−0.78∗	−0.94∗∗∗	−0.86∗∗	−0.73∗		−0.7∗		
Prot_LgtAvg	0.95∗∗∗	0.93∗∗∗		−0.84∗∗	−0.94∗∗∗	−0.99∗∗∗	−0.85∗∗		−0.7∗		
Lacunarity					0.86∗∗	0.7∗			0.7∗		
BranchRate	0.86∗∗	0.83∗∗		−0.79∗	−0.93∗∗∗	−0.91∗∗∗	−0.81∗∗				
End_rate	−0.75∗	−0.73∗		0.82∗∗	0.95∗∗∗	0.85∗∗			0.75∗	0.67∗	
Prot_wdt	−0.77∗	−0.74∗		0.86∗∗	0.93∗∗∗	0.86∗∗	0.68∗				

Statistical analysis: ∗*p* < 0.05, ∗∗*p* < 0.01, ∗∗∗*p* < 0.001.

Relations between microstructural features and dynamic rheology were observed in previous studies (Bernklau et al., 2016; Lucas et al., 2019). An exponential relation of branching and end-point rate with rheological parameters like G′ was reported when varying gluten hydration by changing the flour-to-water ratio (Bernklau et al., 2016). A less interconnected network, i.e. low branching rate, was obtained with excess water which decreased G’. Conversely, a high branching rate was related to high elastic behaviour for doughs prepared with reduced water content compared to a reference dough (Lucas et al., 2019). These results highlight the importance of the plasticizing role of the solvent in the microstructure of the gluten network and its rheological behaviour. The properties of the solvent are described by *Ф*_*w,eff*_, which captures the number of hydrogen bonds available in the solvent to plasticize the gluten, regardless the specific structure of the fibres present.

According to the “Loop and Train” model (Belton, 1999), the loop region is highly susceptible to changes in hydration level. The hydrogen bonds formed between polypeptide chains of proteins could be replaced by protein-water hydrogen bonds upon hydration, allowing sufficient sectional movement of the polypeptide chains (Correa et al., 2014; Nawrocka et al., 2017a, Nawrocka et al., 2017b; Nawrocka et al., 2016a, Nawrocka et al., 2016b). Therefore, changes in the hydrogen bond density of the solvent in the presence of soluble fibres, as described by *Ф*_*w,eff*_, can affect these structural arrangements, similar to what was observed with changes in the flour-to-water ratio (Bernklau et al., 2016; Lucas et al., 2019; Zanoletti et al., 2017). Additionally, the competition for water is well documented as one of the main mechanisms influencing changes in gluten structure in the presence of fibres (Zhou et al., 2021). For soluble fibres the competition for water is described by their hygroscopic properties, i.e. *χ*_*eff*_. Overall, the interplay between *Ф*_*w,eff*_ and *χ*_*eff*_ could explain changes in gluten structure which were reflected in the significant contribution of these two parameters to the rheological properties of wheat dough (Table 3).

### 4.8 Relevance of the effective number of hydrogen bonding site N_OH,s_ available in the fibre structure for interaction with gluten

Compared to the other soluble fibres tested, high M_w_ inulins like IQ and TEX notably enhanced structural features of the gluten network, such as branching rate, and decreased lacunarity. These observations suggested that stable hydrogen bond interactions occurred between these soluble fibres and gluten, driven by their high *N*_*OH,s*_ values, alongside mechanisms previously described by *Ф*_*w,eff*_ and *χ*_*eff*_. High M_w_ inulins are rich in hydroxyl groups which can interact with the polypeptide chains via hydrogen bonds. Specifically, glutenin, characterized by β-like structures stabilized by hydrogen bonds, appears to interact with inulins via hydrogen bonding (Nawrocka et al., 2017a). Supporting this hypothesis (Liu et al., 2016) reported that high M_w_ inulins promote β-sheet formation at the expense of β-turn structures, leading to a denser and more homogeneous gluten network. These results were explained by favoured unfolding and aggregation of glutenin, despite a reduction in disulfide bonds. (Amend and Belitz, 1991) and (Sutton et al., 2003) suggested that glutenin unfolding favours intermolecular cross-links in the gluten network, contributing to a continuous network structure. The formation of β-like structures with hydrogen bonds between glutenins was similarly observed in the presence of inulin and pectin (Nawrocka et al., 2017b). The results of this study align with previous findings, indicating that stable and extensive hydrogen bond interactions between soluble fibres and glutenins require a sufficient number of hydroxyl groups available in the soluble fibre structure, as represented by *N*_*OH,s*_. High *N*_*OH,s*_ values for IQ and TEX (Table 1) may account for the distinctive effects on gluten structure observed, while PDX with lower *N*_*OH,s*_ than IQ despite a similar M_w_, showed different structural impacts on the gluten network. This confirmed that *N*_*OH,s*_ of fibres play a more crucial role in gluten interaction than M_w_ alone. The *N*_*OH,s*_ is dictated by the chemical structure and stereochemistry of the fibres.

While some studies have identified inulins and soluble fibres on gluten surfaces (Li et al., 2017; Liu et al., 2016), it is likely that both chemical interactions (via hydrogen bonds) and physical interactions (via water partitioning) concomitantly contribute to the effect on gluten structure (Zhou et al., 2021). For high M_w_ inulins like IQ and TEX, phase separation can occur at certain concentrations, resulting in a fibre-rich phase that binds water and reduces the effective volume fraction available to gluten and other biopolymers (Renzetti et al., 2021a, Renzetti et al., 2021b). This phase separation effectively increases the concentration of both gluten and inulin in their respective phases (Tolstoguzov, 2007). The resulting partial dehydration of gluten can promote protein conformational changes and localized aggregation, leading to a dense and homogeneous protein networks (Zhou et al., 2021). Due to the high number of hydroxyl groups in high M_w_ inulins, both hydrogen bonds and hydrophobic interactions may form between gluten and fibres at the interface between the continuous protein network and the discontinuous inulin phase. These non-covalent interactions act as physical junctions that contribute to the increased mechanical strength of the dough observed in this study (Nieto-Nieto et al., 2015; Guo et al., 2021).

### 4.9 General description of the mechanisms of soluble fibre-gluten interactions

**Fig. 8:**
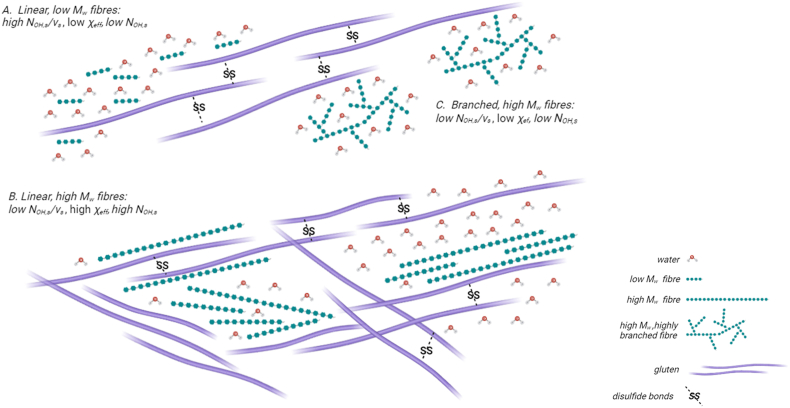
Schematic description of the possible interactions between soluble fibres and gluten depending on the structure and physico-chemical properties of the fibre molecules.

The mechanisms that emerge from this study on the effect of soluble fibre on gluten structure are summarized in Fig. 8. Low M_w_ oligosaccharides possess a high number of hydrogen bonding sites per molar volume, *N*_*OH,s*_*/v*_*s*_, allowing them to effectively plasticize gluten during mixing. These oligosaccharides work synergistically with water, requiring less additional water during flour substitution, despite their high hygroscopic nature, *χ*_*s*_, as depicted in Fig. 8A. However, due to the limited overall *N*_*OH,s*_ in each molecule, these oligosaccharides do not form stable and extensive hydrogen bonds with gluten. Consequently, low M_w_ fibre have a limited effect on gluten structure and rheological behaviour.

In contrast, high M_w_ fibres with linear structures can reduce hydrogen bonding interactions between gluten and solvent because of their low *N*_*OH,s*_*/v*_*s*_. Due to their high volume fraction, fewer water molecules are available to interact with gluten (Fig. 8B), although the low humectant properties result in less water binding. Fibres with high *N*_*OH,s*_ can also form stable and extensive hydrogen bond interactions with gluten (Li et al., 2017). Additionally, linear fibres with high M_w_ tend to self-assemble or entangle, forming phase separated fibre-rich and gluten-rich domains. This phase separation, coupled with hydrogen bonding, promotes a stiff and fine-meshed gluten network characterized by a high branching rate and low lacunarity. On the other hand, high M_w_ fibres that are highly-branched substantially limit gluten hydration due to their affinity for water (low *χ*_*s*_) and low *N*_*OH,s*_*/v*_*s*_ (Fig. 8C). These fibres are also not able to form stable and extensive hydrogen bonds with gluten due to a low *N*_*OH,s*_, nor to entangle or self-aggregate. Consequently, they alter gluten network by reducing its branching rate and promoting aggregation of less hydrated gluten proteins.

## 5 Conclusion

The results of this study indicated that the physico-chemical parameters *Ф*_*w,eff*_ and *χ*_*eff*_ largely modulate phase transitions, thermo-mechanical behaviour during heating and extensional properties of doughs enriched in soluble fibres. The several correlations observed between dough rheology and gluten structural features confirmed the importance of *Ф*_*w,eff*_ and *χ*_*eff*_ in influencing gluten development. These results were obtained for fibres with diverse structural features (i.e. linear vs. branched) and by using fibres individually or in binary mixtures, which suggested a general ability of the proposed principles to describe the effects of complex formulations.

The comparison between inulins and polydextrose with similar M_w_ allowed to gain new insights in the structural features of fibres controlling specific interactions with gluten, beyond the general distinction based on differences in M_w_. Specifically, the promotion of junction zones and branching rate in the gluten network, with concomitant reduction in lacunarity, observed for high M_w_ inulins was driven by high values of effective hydrogen bonding sites *N*_*OH,s*_ available in the fibre molecule. Conversely, polydextrose with low *N*_*OH,s*_ due to a highly branched structure showed opposite effects compared to the inulins, with decreased branching rate and increased lacunarity of the gluten network. The branched structure of polydextrose enhanced its hygroscopic behaviour compared to the linear structure of inulins of comparable M_w_, as indicated by a lower *χ*_*s*_. These findings indicated the importance of fibres structure to effectively interact with gluten by hydrogen bonding interactions. Additionally, high M_w_ inulins may phase separate, further promoting protein aggregation.

The principles outlined in this study could guide the design of fibre mixtures to fine-tune bread dough rheology and gluten structure with fibre-enrichment. For example, high M_w_ fibres like TEX enhanced elastic behaviour (characterized by high G’ and low tan(δ)). The effect of TEX could be compensated by combinations with oligosaccharides like OFP, depending on the specific ratio of the two fibres. This approach could also offset the detrimental effects of fibre-rich by-products, such as bran, by precisely adjusting the properties of the solvent (e.g., water containing soluble fiber). This concept was recently demonstrated in a biscuit application (Renzetti and van der Sman, 2024).

Overall, this study provides new mechanistic insights into the effect of soluble fibres on gluten structure and dough rheology. These insights may support the development and selection of soluble fibres (individual and in mixtures) from diversified sources as functional ingredients in bread-type products, thus benefitting human health and nutrition. Future studies should explore the application of the proposed physico-chemical principles to other soluble fibres sources, such as arabinoxylans.

## CRediT authorship contribution statement

**Stefano Renzetti:** Conceptualization, Methodology, Formal analysis, Visualization, Supervision, Writing - original draft, Writing - review & editing, Funding acquisition. **Lisa Lambertini:** Investigation, Data curation. **Helene C.M. Mocking-Bode:** Investigation, Data curation. **Ruud G.M. van der Sman:** Methodology, Writing - review & editing.

## Declaration of generative AI and AI-assisted technologies in the writing process

During the reviewing process of this work the authors used Grammarly in order to improve the language. After using this tool, the authors reviewed and edited the content as needed and take full responsibility for the content of the publication.

## Declaration of competing interest

Authors declare there is no conflict of interest.

## Data Availability

Data will be made available on request.
