# Supplementary material for: Soluble fibres modulate dough rheology and gluten structure via hydrogen bond density and Flory-Huggins water interaction parameter
Source: Curr Res Food Sci. 2025 Jan 30;10:100991. doi: 10.1016/j.crfs.2025.100991 (PMC11849201; doi:10.1016/j.crfs.2025.100991)
Supplement: Multimedia component 1 [file mmc1.docx]

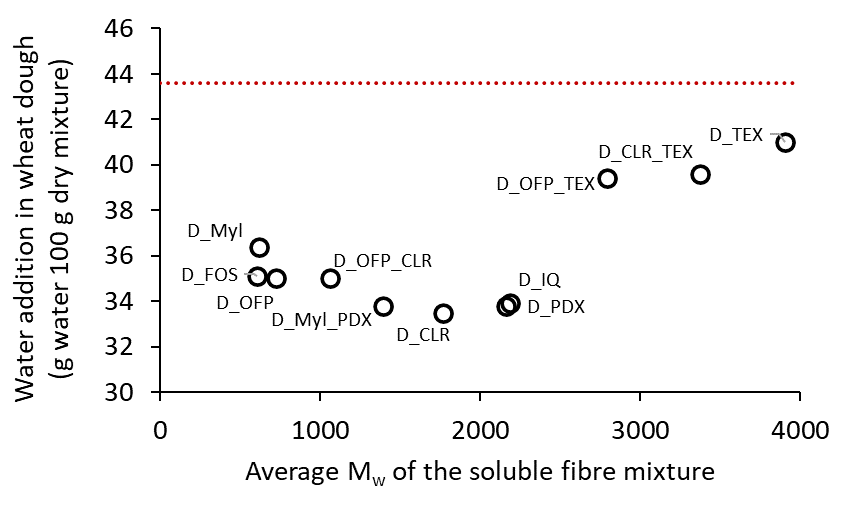


Figure S1. Water additioon level defined with farinograph tests plotted as function of the average molecular weight (M_w_) of the soluble fibres and mixtures thereof. The red dotted line indicates the water addition to the reference dough.


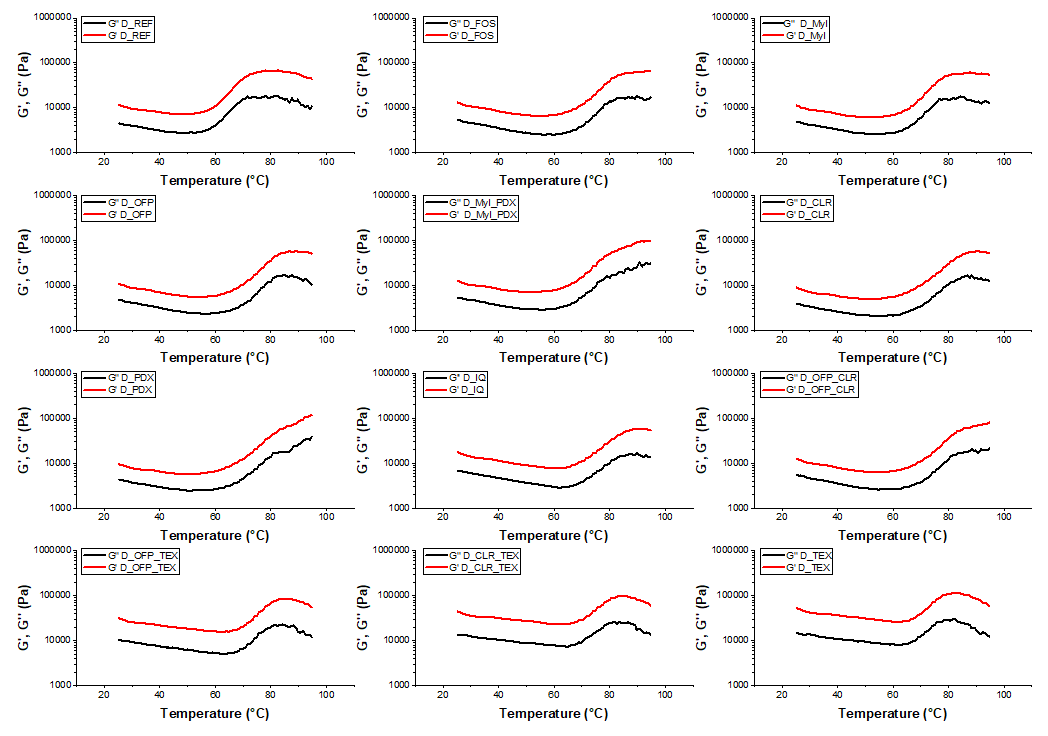


Figure S2. DMTA results for the studied doughs showing G’ and G’’ as function of temperature from 25 to 95 °C.


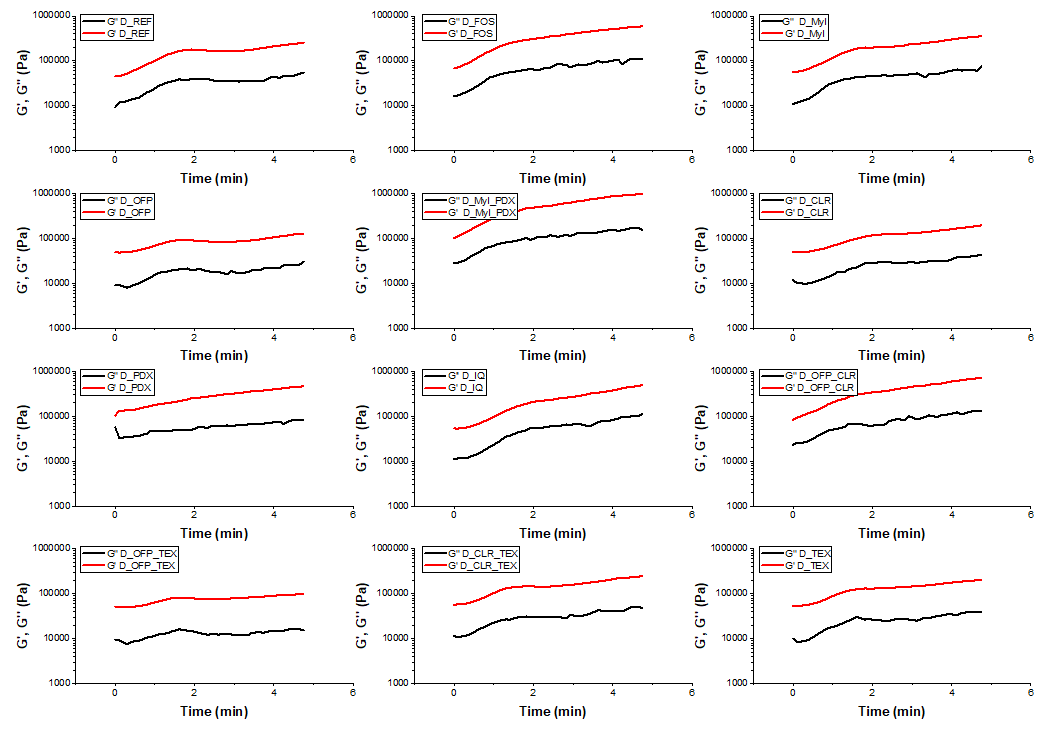


Figure S3. DMTA results showing G’ and G’’ during the 5 min holding time at 95 °C.

Table S1. DSC and DMTA data extracted from dough analysis. Letters in the same colums indicate significant differences.

| Samples | T_on_DSC | T_p_DSC | T_on_DMTA | T_Gmax_ | G’_28C_ | G’_on_ | G’_max_ | G’_95C_ | G’_max_/G’_min_ | tan(δ)_28C_ | tan(δ)_on_ | tan(δ)_95C_ | tan(δ)_Gmax_ |
| --- | --- | --- | --- | --- | --- | --- | --- | --- | --- | --- | --- | --- | --- |
| D_Ref | 63.0b | 90.9g | 58.2f | 82.7c | 9945e | 7027de | 69284b | 183532a | 10.0bcd | 0.417bc | 0.383a | 0.169b | 0.246a |
| D_FOS | 74.3e | 100.4bcd | 66.2bcd | 90.6ab | 10908e | 6476de | 71481b | 306884a | 11.6abcd | 0.441ab | 0.359ab | 0.182ab | 0.252a |
| D_Myl | 68.7cd | 96.1f | 63.0e | 90.5ab | 9626e | 6321de | 83033ab | 575771a | 13.8abcd | 0.439abc | 0.383a | 0.192ab | 0.280a |
| D_OFP | 73.5e | 98.9de | 65.6cd | 91.6a | 8531e | 5387e | 93685ab | 707986a | 18.9ab | 0.453a | 0.373a | 0.193ab | 0.284a |
| D_Myl_PDX | 70.9de | 99.1de | 64.5de | 94.3a | 10164e | 7386de | 109573ab | 899610a | 16.7abc | 0.444ab | 0.369a | 0.175b | 0.312a |
| D_CLR | 74.1e | 101.8ab | 66.2bcd | 92.8a | 8150e | 5610e | 95474ab | 713491a | 18.1ab | 0.453a | 0.359ab | 0.173b | 0.348a |
| D_PDX | 73.8e | 101.3abc | 64.9d | 94.0a | 9162e | 6781de | 137964a | 661223a | 22.8a | 0.459a | 0.365a | 0.175b | 0.328a |
| D_IQ | 73.7 e | 103.3a | 67.8ab | 92.6a | 14830d | 8359d | 90898ab | 298951a | 11.6abcd | 0.411c | 0.354abc | 0.246a | 0.304a |
| D_OFP_CLR | 74.0e | 101.4ab | 66.8abc | 94.5a | 11051e | 6666de | 82994ab | 666367a | 13.2abcd | 0.445ab | 0.354abc | 0.178b | 0.261a |
| D_OFP_TEX | 65.4 bc | 98.9de | 68.3a | 84.6bc | 28090c | 16182c | 86033ab | 100772a | 5.5cd | 0.352d | 0.330bcd | 0.167b | 0.247a |
| D_CLR_TEX | 63.4b | 99.1cde | 68.2a | 82.9c | 39486b | 21744b | 98897ab | 180811a | 4.6d | 0.329de | 0.326cd | 0.182ab | 0.256a |
| D_TEX | 58.3a | 97.397 ef | 67.0abc | 81.281c | 48653a | 28538a | 114947ab | 179475a | 4.2d | 0.300e | 0.308d | 0.187ab | 0.243a |
| *p* | < 0.0001 | < 0.0001 | < 0.0001 | < 0.0001 | < 0.0001 | < 0.0001 | 0.023 | 0.127 | < 0.0001 | < 0.0001 | < 0.0001 | 0.020 | 0.019 |

Table S2. Extensional rheology parameters of the different dough samples. Letters in the same colums indicate significant differences.

| Samples | Resistance to extension (N) | Extensibility (mm) |
| --- | --- | --- |
| D_Ref | 0.64a | 58.8cd |
| D_FOS | 1.36g | 60.6d |
| D_Myl | 1.16 efg | 59.8d |
| D_OFP | 0.89bcd | 69.3e |
| D_Myl_PDX | 1.21fg | 53.2bc |
| D_CLR | 0.96cde | 61.5d |
| D_PDX | 1.24fg | 55.4cd |
| D_IQ | 1.32g | 47.2ab |
| D_OFP_CLR | 1.03def | 72.5e |
| D_OFP_TEX | 0.67a | 58.0cd |
| D_CLR_TEX | 0.75abc | 48.7ab |
| D_TEX | 0.73ab | 43.8a |
| *p* | < 0.0001 | < 0.0001 |
